# Analytic approaches for prognostic studies of persistent pain following knee arthroplasty

**DOI:** 10.2340/17453674.2026.46698

**Published:** 2026-09-07

**Authors:** Daniel L Riddle, Levent Dumenci

**Affiliations:** 1 Distinguished Career Emeritus Professor of Physical Therapy, Orthopaedic Surgery and Rheumatology, Virginia Commonwealth University, Richmond, VA; 2 College of Public Health, Department of Epidemiology and Biostatistics, Temple University, Philadelphia, PA, USA

*Sir—,* Rajamäki and colleagues were interested in identifying preoperative predictors of persistent pain 1-year post-surgery in patients undergoing knee arthroplasty [1]. Given the clinical importance of persistent pain following this typically highly successful surgery, prognostic studies of poor pain outcome are common in the literature [2,3], including a few of our prior publications [4-6].

The investigators chose 3 pain outcomes of interest. The primary outcome was a dichotomized single-item pain severity measure of either moderate or severe pain, derived from the first question in the Oxford Knee Scale (OKS), the second was a dichotomized final score of < 25 for the full OKS (ranging from 0 to 48 with higher scores equating to better outcome), and third was a change of < 8 points for the OKS, less than the minimal clinically important difference (MCID) reported for the OKS [7]. For the MCID outcome, participants had to have a preoperative OKS score of < 28 to reduce ceiling-effect risk. Regarding choice of outcomes, all were derived from the OKS and were therefore not independent. Additionally, dichotomizing all outcomes using arbitrary cut points reduces variability and increases measurement error.

Notably, the extent of missing data for each outcome was also substantial, ranging from 52% to 69%. The investigators indicated they assumed the data was missing completely at random, an assumption that was unlikely given that postsurgical outcome data is more likely to be missing not at random [8]. For example, participants with worse mental health and greater sociodemographic challenges are less likely to provide follow-up measures.

Given the extensive variation in MCID estimates for knee arthroplasty [9,10], and the lack of validity of these approaches [11], we have proposed an alternative prognostic approach: the use of latent growth curve modeling to statistically identify good vs poor pain and function outcome trajectories following knee arthroplasty [4,12]. This method has been externally validated [6], does not rely on arbitrary cut points, accounts for random measurement error, is scientifically falsifiable, and allows for identification of predictors of poor outcome. The investigators did not address this more contemporary and, we believe, superior prognostic approach to estimating the probability of poor pain outcome for patients undergoing knee arthroplasty. Either the latent variable modeling approaches or the latent difference score model [13] is a viable alternative to arithmetic difference score-based anchor-driven MCID calculation with AUCs in the 0.77–0.90 range. The investigators’ reported AUC range of 0.64–0.68 [1] calls for improvements in machine learning algorithms by better handling measurement and methodological issues in predictive modeling.

## Notes

Complete disclosure of interest forms according to ICMJE are available on the article page, doi: 10.2340/17453674.2026.46698

## References

[1] Rajamäki A, Reito A, Karsikas M, Niemeläinen M, Eskelinen A. Predicting persistent pain after total knee arthroplasty using different machine learning algorithms. Acta Orthop. 2026; 97: 423-9. doi: 10.2340/17453674.2026.45965. 42329268 PMC13285364

[2] Cheng H Y, Beswick A D, Bertram W, Siddiqui M A, Gooberman-Hill R, Whitehouse M R, et al. What proportion of people have long-term pain after total hip or knee replacement? An update of a systematic review and meta-analysis. BMJ Open 2025; 15(5): e088975. doi: 10.1136/bmjopen-2024-088975. PMC12096998 40398950

[3] Beswick A D, Wylde V, Gooberman-Hill R, Blom A, Dieppe P. What proportion of patients report long-term pain after total hip or knee replacement for osteoarthritis? A systematic review of prospective studies in unselected patients. BMJ Open 2012; 2(1): e000435. doi: 10.1136/bmjopen-2011-000435. PMC3289991 22357571

[4] Riddle D L, Dumenci L. Using two predictive models to capture two types of poor outcomes in knee arthroplasty: a multisite longitudinal cohort study. Arthritis Rheumatol 2024; 76(7): 1036-46. doi: 10.1002/art.42819. 38327016 PMC11213671

[5] Riddle D L, Dumenci L. Performance of baseline quartile-stratified minimal clinically important difference estimates was superior to individual minimal clinically important difference estimates when compared with a gold standard comparator of important change. Pain 2025; 166(6): 1450-6. doi: 10.1097/j.pain.0000000000003492. 39787274 PMC12074890

[6] Riddle D L, Macfarlane G J, Hamilton D F, Beasley M, Dumenci L. Cross-validation of good versus poor self-reported outcome trajectory types following knee arthroplasty. Osteoarthritis Cartilage 2022; 30(1): 61-8. doi: 10.1016/j.joca.2021.09.004. 34534662

[7] Beard D J, Harris K, Dawson J, Doll H, Murray D W, Carr A J, et al. Meaningful changes for the Oxford hip and knee scores after joint replacement surgery. J Clin Epidemiol 2015; 68(1): 73-9. doi: 10.1016/j.jclinepi.2014.08.009. 25441700 PMC4270450

[8] Ayilara O F, Zhang L, Sajobi T T, Sawatzky R, Bohm E, Lix L M. Impact of missing data on bias and precision when estimating change in patient-reported outcomes from a clinical registry. Health Qual Life Outcomes 2019; 17(1): 106. doi: 10.1186/s12955-019-1181-2. 31221151 PMC6585083

[9] Deckey D G, Verhey J T, Gerhart C R B, Christopher Z K, Spangehl M J, Clarke H D, et al. There are considerable inconsistencies among minimum clinically important differences in TKA: a systematic review. Clin Orthop Relat Res; 2023; 481(1): 63-80. doi: 10.1097/CORR.0000000000002440. 36200846 PMC9750659

[10] MacKay C, Clements N, Wong R, Davis A M. A systematic review of estimates of the minimal clinically important difference and patient acceptable symptom state of the Western Ontario and McMaster Universities Osteoarthritis Index in patients who underwent total hip and total knee replacement. Osteoarthritis Cartilage 2019; 27(10): 1408-19. doi: 10.1016/j.joca.2019.05.002. 31096046

[11] Riddle D L, Dumenci L. Limitations of minimal clinically important difference estimates and potential alternatives. J Bone Joint Surg Am 2024; 06(10): 931-7. doi: 10.2106/JBJS.23.00467. 38060688

[12] Dumenci L, Perera R, Keefe F, Ang D, Slover J, Jensen M, et al. Model-based pain and function outcome trajectory types for patients undergoing knee arthroplasty: a secondary analysis from a randomized clinical trial. Osteoarthritis Cartilage 2019; 27(6): 878-84. doi: 10.1016/j.joca.2019.01.004. 30660721 PMC6536318

[13] Riddle D L, Dumenci L. The latent difference score model is a viable alternative to arithmetic difference score-based anchor-driven minimal important change calculation. J Patient Rep Outcomes 2026; 10(1): 118. doi: 10.1186/s41687-026-01085-2. 42162387 PMC13369083

